# NMFNA: A Non-negative Matrix Factorization Network Analysis Method for Identifying Modules and Characteristic Genes of Pancreatic Cancer

**DOI:** 10.3389/fgene.2021.678642

**Published:** 2021-07-22

**Authors:** Qian Ding, Yan Sun, Junliang Shang, Feng Li, Yuanyuan Zhang, Jin-Xing Liu

**Affiliations:** ^1^School of Computer Science, Qufu Normal University, Rizhao, China; ^2^School of Information and Control Engineering, Qingdao University of Technology, Qingdao, China

**Keywords:** pancreatic cancer, non-negative matrix factorization, module, network analysis, characteristic gene

## Abstract

Pancreatic cancer (PC) is a highly fatal disease, yet its causes remain unclear. Comprehensive analysis of different types of PC genetic data plays a crucial role in understanding its pathogenic mechanisms. Currently, non-negative matrix factorization (NMF)-based methods are widely used for genetic data analysis. Nevertheless, it is a challenge for them to integrate and decompose different types of genetic data simultaneously. In this paper, a non-NMF network analysis method, NMFNA, is proposed, which introduces a graph-regularized constraint to the NMF, for identifying modules and characteristic genes from two-type PC data of methylation (ME) and copy number variation (CNV). Firstly, three PC networks, i.e., ME network, CNV network, and ME–CNV network, are constructed using the Pearson correlation coefficient (PCC). Then, modules are detected from these three PC networks effectively due to the introduced graph-regularized constraint, which is the highlight of the NMFNA. Finally, both gene ontology (GO) and pathway enrichment analyses are performed, and characteristic genes are detected by the multimeasure score, to deeply understand biological functions of PC core modules. Experimental results demonstrated that the NMFNA facilitates the integration and decomposition of two types of PC data simultaneously and can further serve as an alternative method for detecting modules and characteristic genes from multiple genetic data of complex diseases.

## Introduction

Pancreatic cancer (PC) is a highly fatal disease of the digestive system and it is becoming an increasingly common cause of cancer mortality, yet its pathogenic mechanisms remain unclear (Mizrahi et al., 2020). Therefore, comprehensively analyzing multiple types of PC genetic data to understand its pathogenic mechanisms has become a hot topic and many studies have been conducted. For instance, Wu et al. (2011) applied the lasso penalized Cox regression to transcriptome data to identify genes that are directly related to PC survival. Yang et al. (2013) identified thousands of differentially expressed genes of PC and then six genes were predicted to be involved in PC development. Gong et al. (2014) integrated pathway information into PC survival analysis and applied the doubly regularized Cox regression model to microarray data to identify both PC-related genes and pathways. Kwon et al. (2015) used the support vector machine to evaluate the diagnostic performance of PC biomarkers based on miRNA and mRNA expression data. Tao et al. (2016) performed a comprehensive search of electronic literature sources to evaluate the association between *K-ras* gene mutations and PC survival. Li et al. (2018) identified two hub genes of PC from the integrated microarray data and then validated them in RNA-sequencing data by *k*-nearest neighbor and random forest algorithms. These studies provided several underlying biomarkers and can help cancer researchers design new strategies for the early detection and diagnosis of PC (Gong et al., 2014).

Currently, non-negative matrix factorization (NMF)-based methods are widely used for genetic data analysis. For example, Mishra and Guda (2016) applied the NMF to genome-scale methylome analysis of PC data and detected three distinct molecular subtypes. Wang et al. (2013) proposed the maximum correntropy criterion-based NMF (NMF-MCC) method for cancer clustering on gene expression (GE) data. Zhao et al. (2018) used the NMF bi-clustering method to identify subtypes of pancreatic ductal adenocarcinoma, which is the most common type of PC. Xiao et al. (2018) proposed the graph-regularized NMF to discover potential associations between miRNAs and diseases. These methods show that the NMF is a powerful tool for genetic data analysis. Nevertheless, it is a challenge for them to integrate and decompose different types of genetic data simultaneously. Zhang et al. (2012) adopted the joint NMF (jNMF) method to address this challenge, which projects multiple types of genomic data onto a common coordinate system, and applied the jNMF to the methylation (ME), GE, and miRNA expression data of ovarian cancer to identify cancer-related multidimensional modules. Yang and Michailidis (2016) introduced the integrative NMF (iNMF) to analyze multimodal data, which includes a sparsity option for jointly decomposing heterogeneous data, and also evaluated the iNMF on ME, GE, and miRNA expression data of ovarian cancer. These integrated NMF methods can reveal pathogenic mechanisms that would have been overlooked with only a single type of data, and uncover associations between different layers of cellular activities (Zhang et al., 2012).

However, most of these NMF-based methods only consider individual genetic effects and ignore interaction effects among different features. It has been widely accepted that interaction effects could unveil a large portion of unexplained pathogenic mechanisms of cancers (Ding et al., 2019). For capturing these interaction effects, several NMF-based network analysis methods have been proposed due to network facilitating presenting interactions between features. Liu et al. (2014) developed a network-assisted co-clustering algorithm for the identification of cancer subtypes, which first assigns weights to genes based on their impact in the network, and then utilizes the non-negative matrix trifactorization (TriNMF) to cluster cancer patients (Ding et al., 2006). Chen and Zhang adopted the NMF framework in a network manner (NetNMF) to integrate pairwise genomic data for identifying two-level modular patterns and the relationships among these modules (Chen and Zhang, 2018). Elyanow et al. (2020) proposed the netNMF-sc method to cluster cells based on prior knowledge of gene–gene interactions. Nevertheless, the netNMF-sc ignored interaction effects among different features and used the decomposed submatrix to construct the network, which might weaken the internal connection between nodes in the network. Gao et al. (2019) proposed the integrated graph-regularized NMF (iGMFNA) model for clustering and network analysis of cancers, which decomposes the integrated data into submatrices for constructing networks. Zheng et al. (2019) used the NMF to integrate ME and copy number variation (CNV) networks for identifying prognostic biomarkers in ovarian cancer. These NMF-based network analysis methods provide new insights into the pathogenic mechanisms of cancers, especially their interaction effects.

Inspired by both integration and network-assisted strategies of the NMF, in this paper, we presented a NMF network analysis method, NMFNA for short, based on graph-regularized constraint, to identify modules and characteristic genes from integrated ME and CNV data of PC. Firstly, the Pearson correlation coefficient (PCC) is employed to construct three PC networks, i.e., ME network, CNV network, and ME–CNV network. Then, these networks are further integrated and decomposed simultaneously to identify modules effectively due to the introduced graph-regularized constraint, which is the highlight of the NMFNA. Finally, both gene ontology (GO) and pathway enrichment analyses are performed, and characteristic genes are detected by the multimeasure score, to deeply understand biological functions of PC core modules. Experimental results demonstrated that the NMFNA facilitates the integration and decomposition of two types of PC data simultaneously and can further serve as an alternative method for detecting modules and characteristic genes from multiple genetic data of complex diseases.

## Methods

### Non-negative Matrix Factorization Methods

The NMF (Lee and Seung, 1999) and its variants have been increasingly applied to identify modules in biological networks (Chen and Zhang, 2018; Wang et al., 2018; Gao et al., 2019). For a biological network **X**^*m*×*n*^, the NMF can decompose it into two non-negative matrices **U**^*m*×*k*^ and **V**^*k*×*n*^, such that **X** ≈ *V*, where *k* < min (*m*,*n*). The Euclidean distance between **X** and its approximation matrix **UV** is applied to minimize the factorization error, which can be written as,

(1) $$\begin{array}{c} \min_{U,V}||\text{X}-\text{UV}||{}_{F}^{2}\\ \begin{array}{cc} s.t. & \text{U}\geq 0,\text{V}\geq 0 \end{array} \end{array}$$

where $||\cdot ||{}_{F}^{2}$ is the Frobenius norm of a matrix. Since it is difficult to find a global minimal solution by optimizing the convex and non-linear objective function, the NMF adopts the multiplicative iterative update algorithm to approximate **U** and **V**,

(2) $$u_{i\,k}\leftarrow u_{i\,k}\,\frac{{\left({\text{XV}}^{T}\right)}_{i\,k}}{{\left(\text{U}\,V\,V^{T}\right)}_{i\,k}}$$

(3) $$v_{k\,j}\leftarrow v_{k\,j}\,\frac{{\left({\text{U}}^{T}\,\text{X}\right)}_{k\,j}}{{\left({\text{U}}^{T}\,\text{UV}\right)}_{k\,j}}$$

In addition to the two-factor NMF, the three-factor NMF also plays an important role in matrix factorization, which constrains scales of **U** and **V** by an extra factor **S**, i.e., **X** ≈ *USV*. This factored matrix **S** not only provides an additional degree of freedom to make the approximation tight, but also indicates associations between identified modules (Chen and Zhang, 2018). A three-factor NMF variant TriNMF (Ding et al., 2006) is defined as,

(4) $$\begin{array}{c} \min_{U,V}{||\text{X}-\text{USV}||}_{F}^{2}\\ \begin{array}{cc} s.t. & \text{U}\geq 0,\text{S}\geq 0,\text{V}\geq 0 \end{array} \end{array}$$

which minimizes the objective function by,

(5) $$u_{i\,k}\leftarrow u_{i\,k}\,\frac{{\left({\text{XVS}}^{T}\right)}_{i\,k}}{{\left({\text{UU}}^{T}\,{\text{XVS}}^{T}\right)}_{i\,k}}$$

(6) $$v_{k\,j}\leftarrow v_{k\,j}\,\frac{{\left({\text{X}}^{T}\,\text{US}\right)}_{k\,j}}{{\left({\text{VV}}^{T}\,{\text{X}}^{T}\,\text{US}\right)}_{k\,j}}$$

(7) $$s_{k\,k}\leftarrow s_{k\,k}\,\frac{{\left({\text{U}}^{T}\,\text{XV}\right)}_{k\,k}}{{\left({\text{U}}^{T}\,\text{US}\,V^{T}\,V\right)}_{k\,k}}$$

Particularly, if **X** is the symmetric similarity matrix, it could be decomposed into **U***SU*^*T*^. For pairwise biological networks with the same samples but two types of features, ${\text{X}}_{1}^{m_{1}\times n}$ and ${\text{X}}_{2}^{m_{2}\times n}$, combining the idea of two-factor and three-factor NMF, the NetNMF (Chen and Zhang, 2018) is defined as,

(8) $$\begin{array}{c} \min_{G_{1},G_{2},S_{11},S_{22}}{||{\text{R}}_{11}-{\text{G}}_{1}\,{\text{S}}_{11}\,{\text{G}}_{1}^{T}||}_{F}^{2}+\alpha \,{||{\text{R}}_{12}-{\text{G}}_{1}\,{\text{G}}_{2}^{T}||}_{F}^{2}+\beta \,{||{\text{R}}_{22}-{\text{G}}_{2}\,{\text{S}}_{22}\,{\text{G}}_{2}^{T}||}_{F}^{2}\\ \begin{array}{cc} s.t. & {\text{G}}_{1}\geq 0,{\text{G}}_{2}\geq 0,{\text{S}}_{11}\geq 0 \end{array},{\text{S}}_{22}\geq 0 \end{array}$$

where ${\text{R}}_{11}^{m_{1}\times m_{1}}$ and ${\text{R}}_{22}^{m_{2}\times m_{2}}$ are the symmetric feature similarity matrices of **X**_1_ and **X**_2_, respectively, that is, their respective co-expression networks; ${\text{R}}_{12}^{m_{1}\times m_{2}}$ is the two-type feature similarity matrix (co-expression network) between **X**_1_ and **X**_2_; ${\text{G}}_{1}^{m_{1}\times k}$ and ${\text{G}}_{2}^{m_{2}\times k}$ are the non-negative factored matrices used for identifying modules in their respective networks; ${\text{S}}_{11}^{k\times k}$ and ${\text{S}}_{22}^{k\times k}$ are also symmetric matrices whose diagonal elements can be used for measuring associations between identified modules; *k* is the user prespecified dimension parameter; α and β are used to balance three terms of the objective function and default settings are *m*_1_/*m*_2_ and (*m*_1_/*m*_2_)^2^, respectively (Chen and Zhang, 2018). The NetNMF minimizes the objective function by,

(9) $${\left(g_{1}\right)}_{i\,k}\leftarrow {\left(g_{1}\right)}_{i\,k}\,\frac{{\left(2\,{\text{R}}_{11}\,{\text{G}}_{1}\,{\text{S}}_{11}+\alpha \,{\text{R}}_{12}\,{\text{G}}_{2}\right)}_{i\,k}}{{\left(2\,{\text{G}}_{1}\,{\text{S}}_{11}\,{\text{G}}_{1}^{T}\,{\text{G}}_{1}\,{\text{S}}_{11}+\alpha \,{\text{G}}_{1}\,{\text{G}}_{2}^{T}\,{\text{G}}_{2}\right)}_{i\,k}}$$

(10) $${\left(g_{2}\right)}_{k\,j}\leftarrow {\left(g_{2}\right)}_{k\,j}\,\frac{{\left(2\,\beta \,{\text{R}}_{22}\,{\text{G}}_{2}\,{\text{S}}_{22}+\alpha \,{\text{R}}_{12}^{T}\,{\text{G}}_{1}\right)}_{k\,j}}{{\left(2\,\beta \,{\text{G}}_{2}\,{\text{S}}_{22}\,{\text{G}}_{2}^{T}\,{\text{G}}_{2}\,{\text{S}}_{22}+\alpha \,{\text{G}}_{2}\,{\text{G}}_{1}^{T}\,{\text{G}}_{1}\right)}_{k\,j}}$$

(11) $${\left(s_{11}\right)}_{k\,k}\leftarrow {\left(s_{11}\right)}_{k\,k}\,\frac{{\left({\text{G}}_{1}^{T}\,{\text{R}}_{11}\,{\text{G}}_{1}\right)}_{k\,k}}{{\left({\text{G}}_{1}^{T}\,{\text{G}}_{1}\,{\text{S}}_{11}\,{\text{G}}_{1}^{T}\,{\text{G}}_{1}\right)}_{k\,k}}$$

(12) $${\left(s_{22}\right)}_{k\,k}\leftarrow {\left(s_{22}\right)}_{k\,k}\,\frac{{\left({\text{G}}_{2}^{T}\,{\text{R}}_{22}\,{\text{G}}_{2}\right)}_{k\,k}}{{\left({\text{G}}_{2}^{T}\,{\text{G}}_{2}\,{\text{S}}_{22}\,{\text{G}}_{2}^{T}\,{\text{G}}_{2}\right)}_{k\,k}}$$

### Non-negative Matrix Factorization Network Analysis Method

In order to further improve the capability of identifying modules and capturing interaction effects, we proposed the NMFNA method by introducing the graph-regularized constraint into the NetNMF, which can preserve the inherent geometrical structure of input networks (Deng et al., 2011). For demonstrating its effectiveness, in the study, we applied the NMFNA to two-type PC data of ME and CNV to identify modules and characteristic genes. In fact, the NMFNA is universally useful and can be applied to any type of genetic data in various complex diseases.

**FIGURE 1:**
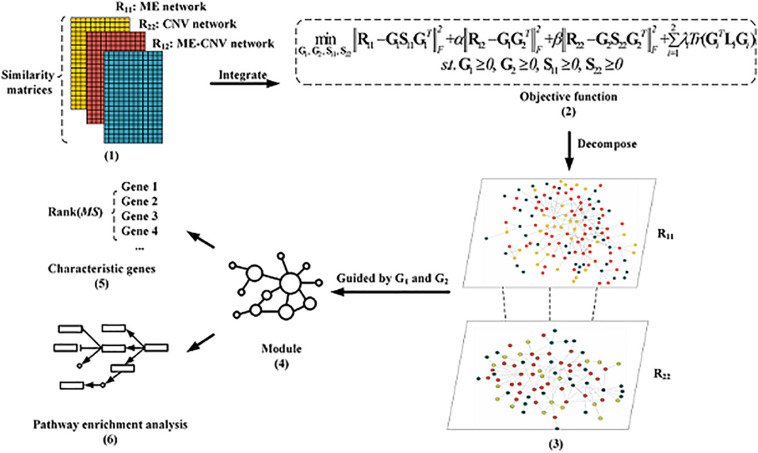
The overall workflow of the non-negative matrix factorization network analysis (NMFNA) for identifying modules and characteristic genes by integrating methylation (ME) and copy number variation (CNV) data of pancreatic cancer (PC).

The overall workflow of the NMFNA for identifying modules and characteristic genes by integrating ME and CNV data of PC is shown in Figure 1. It is seen that the NMFNA mainly has three stages. In the first stage, three co-expression networks are constructed from ME and CNV data of PC. In the second stage, these three networks are applied to the objective function to identify modules. In the third stage, both GO and pathway enrichment analyses are performed, and characteristic genes are detected, to deeply understand biological functions of PC core modules. Among them, the objective function, which introduces the graph-regularized constraint, is the highlight of the NMFNA.

The graph-regularized constraint indicates the inherent geometrical structure of the input networks. In other words, the graph-regularized constraint ensures that interactive features in the Euclidean space are also close to each other in the low-dimensional space, which is defined as,

(13) $$G\,R\,C=\frac{1}{2}\,\sum_{i\,j}{||v_{i}-v_{j}||}^{2}\,{\text{Z}}_{i\,j}=T\,r\,\left({\text{G}}^{T}\,\text{LG}\right)$$

where **Z** is the sparse weight matrix established using the geometrical information of **X** (Xiao et al., 2018), and **Z***ij* represents the similarity between gene *v*_*i*_ and *v*_*j*_. *Tr*(⋅) represents the trace of a matrix, and **G** is the factored matrix of the biological network **X** by the NMF. Define a diagonal matrix **D** whose elements are column sums of matrix Z, that is, **D**_*ii*_ = ∑_*i*_ **Z**_*ij*_, and **L** is the graph Laplacian matrix defined as:

(14) L=D-Z.

Based on the NetNMF and the graph-regularized constraint, the objective function of the NMFNA is defined as,

(15) $$\begin{array}{c} \min_{G_{1},G_{2},S_{11},S_{22}}{||{\text{R}}_{11}-{\text{G}}_{1}\,{\text{S}}_{11}\,{\text{G}}_{1}^{T}||}_{F}^{2}+\alpha \,{||{\text{R}}_{12}-{\text{G}}_{1}\,{\text{G}}_{2}^{T}||}_{F}^{2}+\\ \beta \,{||{\text{R}}_{22}-{\text{G}}_{2}\,{\text{S}}_{22}\,{\text{G}}_{2}^{T}||}_{F}^{2}+\sum_{i=1}^{2}\lambda_{i}\,T\,r\,\left({\text{G}}_{i}^{T}\,{\text{L}}_{i}\,{\text{G}}_{i}\right)\\ \begin{array}{cc} s.t. & {\text{G}}_{1}\geq 0,{\text{G}}_{2}\geq 0,{\text{S}}_{11}\geq 0 \end{array},{\text{S}}_{22}\geq 0 \end{array}$$

where **R**_11_ and **R**_22_ are the ME and CNV co-expression networks, **R**_12_ is the ME–CNV co-expression network, λ is the tuning parameter to adjust the closeness between interactive features, and other notation meanings and parameter settings are the same as those in the NetNMF.

The multiplicative iterative update algorithm is adopted here to minimize the objective function of the NMFNA. Suppose **ψ**_1_, **ψ**_2_, **ψ**_3_, and **ψ**_4_ are matrices of Lagrange multipliers that, respectively, constrain **S**_11_ ≥ 0, **S**_22_ ≥ 0, **G**_1_ ≥ 0, and **G**_2_ ≥ 0, the Lagrange function *f* of the NMFNA is,

(16) $$\begin{array}{c} f=t\,r\,\left({\left({\text{R}}_{11}-{\text{G}}_{1}\,{\text{S}}_{11}\,{\text{G}}_{1}^{T}\right)}^{T}\,\left({\text{R}}_{11}-{\text{G}}_{1}\,{\text{S}}_{11}\,{\text{G}}_{1}^{T}\right)\right)+\\ \alpha \,t\,r\,\left({\left({\text{R}}_{12}-{\text{G}}_{1}\,{\text{G}}_{2}^{T}\right)}^{T}\,\left({\text{R}}_{12}-{\text{G}}_{1}\,{\text{G}}_{2}^{T}\right)\right)\\ +\beta \,t\,r\,\left({\left({\text{R}}_{22}-{\text{G}}_{2}\,{\text{S}}_{22}\,{\text{G}}_{2}^{T}\right)}^{T}\,\left({\text{R}}_{22}-{\text{G}}_{2}\,{\text{S}}_{22}\,{\text{G}}_{2}^{T}\right)\right)+\\ \lambda_{1}\,T\,r\,\left({\text{G}}_{1}^{T}\,{\text{L}}_{1}\,{\text{G}}_{1}\right)+\lambda_{2}\,T\,r\,\left({\text{G}}_{2}^{T}\,{\text{L}}_{2}\,{\text{G}}_{2}\right)\\ +t\,r\,\left(\psi_{1}^{T}\,{\text{S}}_{11}\right)+t\,r\,\left(\psi_{2}^{T}\,{\text{S}}_{22}\right)+t\,r\,\left(\psi_{3}^{T}\,{\text{G}}_{1}\right)+t\,r\,\left(\psi_{4}^{T}\,{\text{G}}_{2}\right) \end{array}$$

Hence, partial derivatives of **f** with respect to **S**_11_, **S**_22_, **G**_1_, and **G**_2_ are,

(17) $$\frac{\partial f}{\partial {\text{S}}_{11}}=-2\,{\text{G}}_{1}^{T}\,{\text{R}}_{11}\,{\text{G}}_{1}+2\,{\text{G}}_{1}^{T}\,{\text{G}}_{1}\,{\text{S}}_{11}\,{\text{G}}_{1}^{T}\,{\text{G}}_{1}+\psi_{1}$$

(18) $$\frac{\partial f}{\partial {\text{S}}_{22}}=-2\,{\text{G}}_{2}^{T}\,{\text{R}}_{22}\,{\text{G}}_{2}+2\,{\text{G}}_{2}^{T}\,{\text{G}}_{2}\,{\text{S}}_{22}\,{\text{G}}_{2}^{T}\,{\text{G}}_{2}+\psi_{2}$$

$$\frac{\partial f}{\partial {\text{G}}_{1}}=4\,\left({\text{G}}_{1}\,{\text{S}}_{11}\,{\text{G}}_{1}^{T}\,{\text{G}}_{1}\,{\text{S}}_{11}-{\text{R}}_{11}\,{\text{G}}_{1}\,{\text{S}}_{11}\right)+2\,\alpha$$

(19) $$\left({\text{G}}_{1}\,{\text{G}}_{2}^{T}\,{\text{G}}_{2}-{\text{R}}_{12}\,{\text{G}}_{2}\right)+2\,\lambda_{1}\,{\text{L}}_{1}\,{\text{G}}_{1}+\psi_{3}$$

$$\frac{\partial f}{\partial {\text{G}}_{2}}=4\,\beta \,\left({\text{G}}_{2}\,{\text{S}}_{22}\,{\text{G}}_{2}^{T}\,{\text{G}}_{2}\,{\text{S}}_{22}-{\text{R}}_{22}\,{\text{G}}_{2}\,{\text{S}}_{22}\right)+2\,\alpha$$

(20) $$\left({\text{G}}_{2}\,{\text{G}}_{1}^{T}\,{\text{G}}_{1}-{\text{R}}_{12}\,{\text{G}}_{1}\right)+2\,\lambda_{2}\,{\text{L}}_{2}\,{\text{G}}_{2}+\psi_{4}$$

According to Karush–Kuhn–Tucher conditions (Dreves et al., 2011), i.e., **ψ**_1_**S**_11_ = 0, **ψ**_2_**S**_22_ = 0, **ψ**_3_**G**_1_ = 0, and **ψ**_4_**G**_2_ = 0, iterative formulas can be written as,

(21) $${\left(s_{11}\right)}_{k\,k}\leftarrow {\left(s_{11}\right)}_{k\,k}\,\frac{{\left({\text{G}}_{1}^{T}\,{\text{R}}_{11}\,{\text{G}}_{1}\right)}_{k\,k}}{{\left({\text{G}}_{1}^{T}\,{\text{G}}_{1}\,{\text{S}}_{11}\,{\text{G}}_{1}^{T}\,{\text{G}}_{1}\right)}_{k\,k}}$$

(22) $${\left(s_{22}\right)}_{k\,k}\leftarrow {\left(s_{22}\right)}_{k\,k}\,\frac{{\left({\text{G}}_{2}^{T}\,{\text{R}}_{22}\,{\text{G}}_{2}\right)}_{k\,k}}{{\left({\text{G}}_{2}^{T}\,{\text{G}}_{2}\,{\text{S}}_{22}\,{\text{G}}_{2}^{T}\,{\text{G}}_{2}\right)}_{k\,k}}$$

(23) $${\left(g_{1}\right)}_{i\,k}\leftarrow {\left(g_{1}\right)}_{i\,k}\,\frac{{\left(\alpha \,{\text{R}}_{12}\,G_{2}+2\,{\text{R}}_{11}\,{\text{G}}_{1}\,{\text{S}}_{11}+2\,\lambda_{1}\,{\text{Z}}_{1}\,{\text{G}}_{1}\right)}_{i\,k}}{{\left(2\,{\text{G}}_{1}\,{\text{S}}_{11}\,{\text{G}}_{1}^{T}\,{\text{G}}_{1}\,{\text{S}}_{11}+\alpha \,{\text{G}}_{1}\,{\text{G}}_{2}^{T}\,{\text{G}}_{2}+2\,\lambda_{1}\,{\text{D}}_{1}\,{\text{G}}_{1}\right)}_{i\,k}}$$

(24) $${\left(g_{2}\right)}_{k\,j}\leftarrow {\left(g_{2}\right)}_{k\,j}\,\frac{{\left(\alpha \,{\text{R}}_{12}^{T}\,{\text{G}}_{1}+2\,\beta \,{\text{R}}_{22}\,{\text{G}}_{2}\,{\text{S}}_{22}+\lambda_{2}\,{\text{Z}}_{2}\,{\text{G}}_{2}\right)}_{k\,j}}{{\left(2\,\beta \,{\text{G}}_{2}\,{\text{S}}_{22}\,{\text{G}}_{2}^{T}\,{\text{G}}_{2}\,{\text{S}}_{22}+\alpha \,{\text{G}}_{2}\,{\text{G}}_{1}^{T}\,{\text{G}}_{1}+\lambda_{2}\,{\text{D}}_{2}\,{\text{G}}_{2}\right)}_{k\,j}}$$

Two types of modules, namely, ME modules and CNV modules, can be identified from **R**_11_ and **R**_22_ guided by **G**_1_ and **G**_2_, respectively. In particular, **G**_1_ and **G**_2_ are first z-score normalized; then for each column (1,⋯,*k*) of them, those genes whose corresponding weights are greater than or equal to the threshold are considered as a cluster; finally, according to these clusters, subnetworks of **R**_11_ and **R**_22_ can be captured as ME modules and CNV modules. Here, the threshold is set to be 2 according to the reference (Chen and Zhang, 2018). In addition, similar to previous studies (Hou et al., 2019; Zhao et al., 2020), modules with the most genes are known as core modules.

To identify characteristic genes from core modules, which may play an important role in deeply understanding the biological functions of modules, we employ the multimeasure score (MS) to numerically quantify the importance of each gene, which is defined as,

(25) $$M\,S\,\left(v\right)=\frac{D\,C\,\left(v\right)\cdot B\,C\,\left(v\right)}{E\,C\,\left(v\right)}$$

where *DC*(*v*), *BC*(*v*), and *EC*(*v*) are the degree centrality, betweenness centrality, and eccentricity centrality of gene *v* in ${\text{R}}_{11}^{\prime }$ and ${\text{R}}_{22}^{\prime }$, which are networks filtered from **R**_11_ and **R**_22_, respectively, with edge weights higher than a given threshold. Betweenness centrality and eccentricity centrality focus on the global feature of a gene in the network, while degree centrality focuses on the local feature of a gene in the network (Shang et al., 2019); hence, the MS combines both global and local features of a gene.

## Results and Discussion

### Data and Parameter Settings

Two types of PC data, i.e., ME data and CNV data, are downloaded from the TCGA database^1^. These two data have the same samples (176 tumor samples and 4 normal samples) but different features: 21,031 methylations in ME data and 23,627 CNVs in CNV data. Based on these PC data, three co-expression networks, i.e., the ME network ${\text{R}}_{11}^{21,031\times 21,031}$, the CNV network ${\text{R}}_{22}^{23,627\times 23,627}$, and the ME–CNV network ${\text{R}}_{12}^{21,031\times 23,627}$, are constructed using the PCC. Besides, to further prove the experimental results of two types of PC data, we also analyze the GEO datasets of PC. Four profile datasets, i.e., GSE62452, GSE15471, GSE16515, and GSE28735, of PC are downloaded from the GEO database^2^ for this study. Details of these four datasets are shown in Table 1.

**TABLE 1:** Details of GEO datasets of PC.

**Datasets**	**Normal**	**Tumor**	**Genes**
GSE62452	61	69	20,358
GSE15471	36	36	22,188
GSE16515	16	36	22,187
GSE28735	45	45	20,314

**FIGURE 2:**
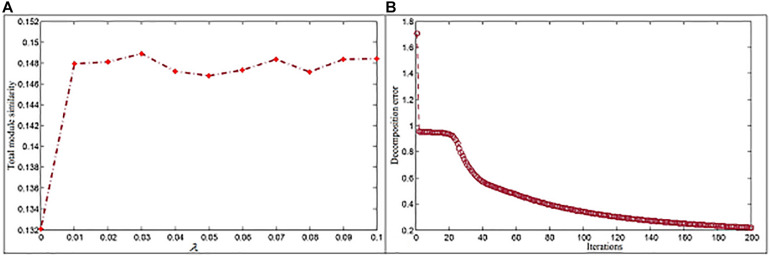
Parameter settings for the NMFNA. **(A)** Is the influence of parameter and **(B)** is the convergence analysis of NMFNA.

In the NMFNA, four parameters should be set, which is, tuning parameters λ_*1*_ and λ_*2*_, the dimension parameter *k*, and the iteration number. λ reflects the degree of imposed graph-regularized constraint. A large one focuses on reaching consensus across views, while a small one cannot tolerate matrix factorization error (Liu et al., 2013). Since these different items have no distinction of importance, and the dimension reduction parameter *k* has a greater impact compared with parameter λ, considering the convenience of comparison, both λ_*1*_ and λ_*2*_ are set to be the same value. We run the NMFNA with different λ values ranging from 0 to 0.1 to select the proper one based on the measure of total module similarity (Wang et al., 2018), which is defined as,

(26) $$T\,M\,S=\sum_{x,y}\frac{\left|M_{x}\,⋂M_{y}\right|}{\min\left(\left|M_{x}\right|,\left|M_{y}\right|\right)}$$

where *M*_*x*_ represents members in module *x*. According to experiment results (Figure 2A), λ_*1*_ and λ_*2*_ are set to be 0.03. The dimension parameter *k* is determined by the singular value decomposition method (Qiao, 2015), and its first inflection point, i.e., 6,834, is selected to *k*. In order to reduce the decomposition error, a large iteration number is used. In the study, we set it to 200 since the decomposition error here has already reached a relatively stable state (Figure 2B).

### GO and Pathway Enrichment Analyses

**FIGURE 3:**
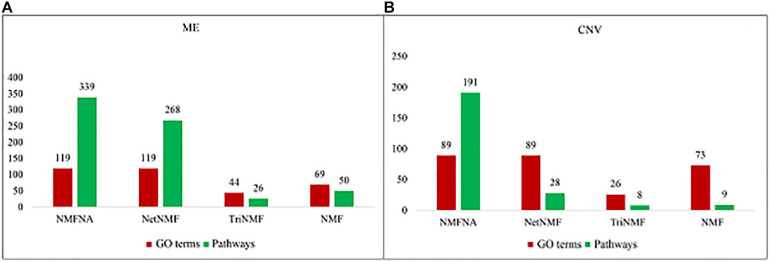
Numbers of GO terms and pathways obtained from enrichment analyses of ME and CNV core modules that identified by compared methods. **(A)** Represents the details of the ME core module and **(B)** represents the details of the CNV core module.

To demonstrate the validity of the NMFNA, we compared it with the NMF, TriNMF, and NetNMF by performing GO and pathway enrichment analyses on their respective identified core modules. The GO enrichment analysis was carried out by an online tool, DAVID Bioinformatics Resources^3^ (Dennis et al., 2003). The pathway enrichment analysis was conducted using the KOBAS v3.0 web server^4^ (Kanehisa et al., 2016), in which, the KEGG pathway, BioCyc, PANTHER, and Reactome databases were used. The numbers of GO terms and pathways (*p*-value < 0.05) obtained from enrichment analyses of ME and CNV core modules identified by the compared methods are shown in Figure 3. It is seen that either ME or CNV core modules identified by the NMFNA have more GO terms and pathways than those identified by other methods, implying that modules identified by the NMFNA might contain more biological information related to understanding the pathogenic mechanisms of PC.

**FIGURE 4:**
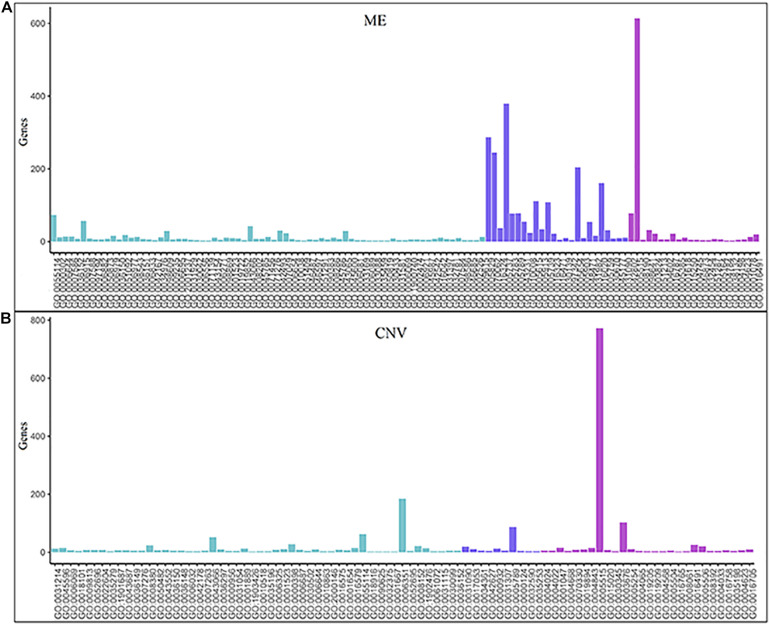
Numbers of enriched genes in GO terms obtained from enrichment analyses of ME and CNV core modules identified by the NMFNA. **(A)** Represents numbers of the enriched gene of the ME core module and **(B)** represents numbers of the enriched gene of the CNV core module.

The numbers of enriched genes in GO terms obtained from the enrichment analyses of ME and CNV core modules identified by the NMFNA are shown in Figure 4. It is seen that for the ME core module, genes are mainly enriched in GO:0005515, GO:0005737, GO:0005829, GO:0070062, GO:0005654, GO:0016020, GO:0005615, and GO:0005739, corresponding to protein binding, cytoplasm, cytosol, extracellular exosome, nucleoplasm, membrane, extracellular space, and mitochondrion; for the CNV core module, genes are mainly enriched in GO:0005515, GO:0006351, GO:0003676, and GO:0005789, corresponding to protein binding, DNA-templated, DNA binding, and endoplasmic reticulum. Details of these significant GO terms are listed in Table 2. Several studies have confirmed that these GO terms contribute to the development of PC cells. The protein binding (GO:0005515) is the most significantly enriched GO term among molecular functions in both ME and CNV core modules. As one of the specific binding protein, IGF binding protein-1 has been confirmed to inhibit the activity of insulin-like growth factor I, which has growth-promoting effects on PC cells (Wolpin et al., 2007). The cytoplasm (GO:0005737) plays an important role in the development of PC by regulating the expression of carbonic anhydrase IX (Juhász et al., 2003). As a member of the cadherin/catenin family, p120(ctn) has been found in the cytosol (GO:0005829) of PC cells (Mayerle et al., 2003), which is correlated to the degree of tumor dedifferentiation. The fractional volume of the extracellular space (GO:0005615) in the PC tissue has been reported to be statistically larger than that in the normal tissue (Yao et al., 2012). The novel mitochondrion interfering compound NPC-26 may effectively inhibit the growth of PC cells by destroying the mitochondria (GO:0005739) (Dong et al., 2016). Genes involved in the DNA-templated (GO:0006351) have been clinically used for treating lung cancer (Lu et al., 2016), which might be speculated to affect other cancers by their pan-cancer co-regulation mechanisms. The nicotine can induce the inhibitor of the DNA binding (GO:0003676) and has been confirmed as an established risk factor for PC (Treviño et al., 2011). The endoplasmic reticulum (GO:0005789) has been identified as the key target in PC, which shows its potential for antitumor drug development (Gajate et al., 2012). Other three GO terms, including extracellular exosome (GO:0070062), nucleoplasm (GO:0005654), and membrane (GO:0016020) also have been reported to associate with PC (Sakai et al., 2019; Zhou et al., 2019).

**TABLE 2:** Details of significant GO terms obtained from enrichment analyses of ME and CNV core modules identified by the NMFNA. adj.P is the *p*-value corrected by the FDR.

**Module**	**ID**	**Name**	**Count**	***p*-value**	**adj.P**
ME, CNV	GO:0005515	Protein binding	614	1.62E-04	4.80E-02
ME	GO:0005737	Cytoplasm	379	3.26E-04	6.93E-07
ME	GO:0005829	Cytosol	287	1.15E-09	5.25E-06
ME	GO:0070062	Extracellular exosome	245	1.74E-08	2.85E-01
ME	GO:0005654	Nucleoplasm	204	8.85E-03	4.77E-01
ME	GO:0016020	Membrane	161	2.13E-02	1.32E-01
ME	GO:0005615	Extracellular space	111	2.10E-03	2.01E-01
ME	GO:0005739	Mitochondrion	108	4.09E-03	9.85E-01
CNV	GO:0006351	Transcription, DNA-templated	185	3.75E-02	7.27E-01
CNV	GO:0003676	Nucleic acid binding	103	1.08E-02	9.26E-01
CNV	GO:0005789	Endoplasmic reticulum membrane	87	2.79E-02	9.53E-02

*ME, methylation; CNV, copy number variation.*

**FIGURE 5:**
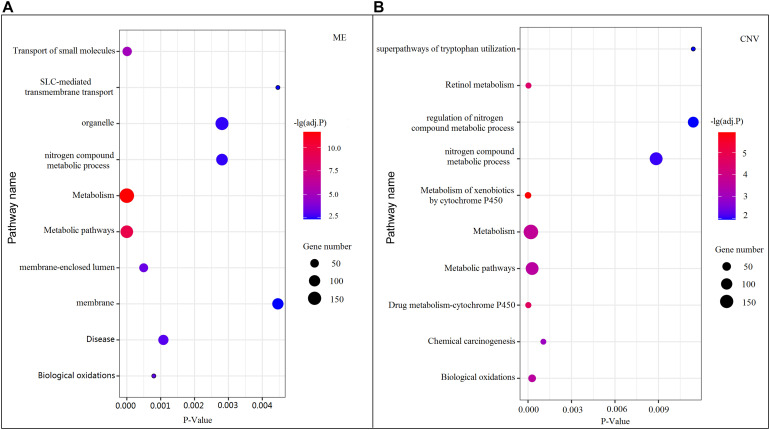
Top 10 pathways enriched in ME and CNV core modules identified by the NMFNA. **(A)** Represents pathways enriched in the ME core module and **(B)** represents pathways enriched in the CNV core module.

Among all pathways obtained from enrichment analyses of ME and CNV core modules identified by the compared methods (Figure 3), we recorded common pathways that appear in at least three out of four methods in Table 3. It is seen that in terms of *p*-values, as well as *p*-values corrected by the false discovery rate (FDR), namely adj.P, the NMFNA performs best among all compared methods, implying that pathways enriched in core modules identified by the NMFNA are more significant than those enriched in core modules identified by other compared methods. To further analyze these core modules, the top 10 pathways according to their adj.P enriched in ME and CNV core modules identified by the NMFNA are listed in Figure 5, in which, the node size and color represent the number of genes enriched in the pathway and the significance of the pathway, respectively. Specifically, two pathways, i.e., transport of small molecules and arachidonic acid metabolism, have already been reported to be associated with PC. The former can filter downregulated differentially expressed genes of PC, while the latter can promote the progress of PC (Biswas et al., 2014; Long et al., 2016). To aid early diagnosis, the metabolism pathway can be used individually or in combination to differentiate people with and without PC. The metabolism of xenobiotics by cytochrome P450 pathway has been considered as an important pathway associated with the progression of cancer (Hu and Chen, 2012). Besides, three other metabolism-related pathways, namely, lipid metabolism and autophagy, glutamine-regulatory enzymes, and Akt/c-Myc pathway (Dando et al., 2013; Blum and Kloog, 2014), have been identified to directly affect the growth of PC cells. The small molecule metabolic process also has been found as the enriched pathway for the biological process of PC (Hu et al., 2017). The identification of immune system-related regulation pathways has been reported to provide several new insights for PC treatment and prognosis (Yang and Michailidis, 2016).

**TABLE 3:** Common pathways appearing in at least three out of four methods.

**Pathways**	**NMFNA**		**NetNMF**		**TriNMF**		**NMF**	
	***p*-value**	**adj.P**	***p*-value**	**adj.P**	***p*-value**	**adj.P**	***p*-value**	**adj.P**
Metabolism	4.58E-16	2.30E-12	3.65E-15	1.86E-11	4.94E-06	1.19E-02	7.70E-04	1.87E-01
Immune system	1.11E-05	4.46E-03	7.72E-06	3.93E-03	1.15E-03	1.33E-01	1.02E-03	1.87E-01
Disease	1.29E-06	1.08E-03	5.27E-04	4.71E-02	1.05E-04	6.63E-02	\	\
Transport of small molecules	6.46E-09	1.08E-05	2.42E-07	3.08E-04	\	\	4.61E-03	3.56E-01
Arachidonic acid metabolism	6.79E-04	5.08E-02	4.62E-03	1.52E-01	4.38E-03	2.13E-01	\	\
Metabolic process	1.27E-04	4.14E-02	4.28E-04	1.26E-01	4.65E-04	4.79E-02	\	\

*adj.P is the *p*-value corrected by the FDR. “\” represents that the pathway cannot be obtained from enrichment analyses of ME and CNV core modules that were identified by the corresponding method.*

### Analysis of Characteristic Genes

In order to further demonstrate the validity of the NMFNA and deeply understand biological functions of core modules, we detect and analyze characteristic genes from these core modules. First, ME and CNV core modules identified by four compared methods are filtered by removing weak edges with their PCC less than or equal to 0.8. Second, based on these filtered networks, the MS of each gene in the corresponding module is calculated, which can be considered as its contribution to interactions among genes in the module. Third, genes in each core module are sorted in descending order according to the MS, and the top ones are viewed as characteristic genes. In the study, the top 10, 30, and 50 characteristic genes in each core module are, respectively, selected and sent to GeneCards^5^ to measure their relevance scores, which represent association strengths between corresponding genes and PC. After that, for each compared method, relevance scores of characteristic genes in both ME and CNV core modules are summated together and recorded in Table 4. It is seen that in all scenarios, scores of the NMFNA are significantly larger than the scores of other compared methods, which implies that characteristic genes detected by the NMFNA are more relevant to PC and are more likely to reveal the pathogenic mechanisms of PC.

**TABLE 4:** Summated relevance scores of characteristic genes in both ME and CNV core modules identified by each compared method.

**Method**	**Top 10**	**Top 30**	**Top 50**
NMFNA	32.80	90.74	220.54
NetNMF	22.51	58.62	97.70
TriNMF	12.03	52.87	76.29
NMF	12.83	81.05	117.23

In addition, for each compared method, PC-related genes in the top 10 characteristic genes of both ME and CNV core modules are retrieved from GeneCards and listed in Table 5. It is seen that the NMFNA hits eight genes and is the winner of all compared methods. We then analyzed in detail the biological functions of these PC-related genes. The *TNKS1BP1* has been reported to regulate cancer cell invasion, which might further affect the progression of PC (Mayerle et al., 2003). The *TK1* level is upregulated 4-fold in the mice PC specimen (Yao et al., 2012); therefore, we naturally speculate that it might also play a potential role in the human PC. The variation of the *KCNJ1* has been claimed to be associated with diabetes (Farook et al., 2002), which is a closely related disease to PC and is generally thought of as the important risk factor of PC. As an independent prognostic biomarker, the *SDC1* has been confirmed to be upregulated in PC (Juuti et al., 2005). Since the *SDC1* is an important paralog of the *SDC3*, we infer that the *SDC3* might be related to PC. The *NR3C2* has been identified as the target of miR-135b-5p, which promotes migration and invasion of PC cells (Zhang et al., 2017). Though the *TTC21A*, *USF1*, and *MAN1C1* have been marked as PC-related genes in GeneCards, and they are indeed associated with several PC complicating diseases, including hyperlipidemia and alcohol-induced mental disorder, there are few supporting literature studies.

**TABLE 5:** PC-related genes in the top 10 characteristic genes of both ME and CNV core modules.

**Method**	**Count**	**Gene**
NMFNA	8	*TTC21A TNKS1BP1 TK1 KCNJ1 USF1 SDC3 MAN1C1 NR3C2*
NetNMF	6	*YTHDF1 TK1 KCNJ1 USF1 CALCOCO1 LIMD1*
TriNMF	5	*MTF1 LTBP4 SDC3 NEDD4 ASCC2*
NMF	5	*UBL4B FCGR1A KMT2D ANK1 PARG*

Besides, the experiments of the GEO datasets are also performed to further verify the effectiveness of modules and characteristic genes identified by the NMFNA method. Firstly, we calculated the numbers of common genes of the four datasets GSE62452, GSE15471, GSE16515, and GSE28735. As shown in Supplementary Figure 1, there are 17,327 common genes, which are more likely to be related to PC. Secondly, Table 6 lists the number of genes in the modules obtained by different methods that overlap with these common genes. It can be seen from Table 6 that genes of the core modules identified by the NMFNA method contain the largest number of common genes, which indicates that these core modules have been verified to be related to PC in different databases.

**TABLE 6:** Numbers of common genes in ME and CNV core modules.

**Module**	**NMFNA**	**NetNMF**	**TriNMF**	**NMF**
ME	1,139	1,135	1,058	1,147
CNV	1,470	1,435	1,019	819

## Conclusion

Pancreatic cancer is a disease with a poor prognosis, in which malignant cells originate in the pancreatic tissue. To understand its pathogenic mechanisms, in this study, based on NMF and graph-regularized constraint, we presented NMFNA to identify modules and characteristic genes from integrated ME and CNV data of PC. First, the ME network, CNV network, and ME–CNV network are constructed by the PCC. Then, these networks are further integrated and decomposed simultaneously to identify modules effectively due to the introduced graph-regularized constraint, which is the highlight of the NMFNA. Finally, both GO and pathway enrichment analyses are performed, and characteristic genes are detected by the multimeasure score, to deeply understand the biological functions of PC core modules. Compared with the NMF, TriNMF, and NetNMF, the NMFNA identified more PC-related GO terms, pathways, and characteristic genes in core modules, demonstrating that the NMFNA facilitates the integration and decomposition of two types of PC data simultaneously and can further serve as an alternative method for detecting modules and characteristic genes from multiple genetic data of complex diseases.

The NMFNA has several advantages. First, it performs well in integrating and decomposing different types of genetic data simultaneously. Second, introducing the graph-regularized constraint into the NMFNA eases the heterogeneity of multiple networks, which is beneficial to detect core modules. Third, the NMFNA can not only consider individual genetic effects but also capture interaction effects among different features contributing to the development of PC. Nevertheless, it still has some limitations. For instance, associations between ME modules and CNV modules, i.e., *S*_*11*_, *S*_*22*_, are not deeply analyzed in theory and experiment; it only supports the integration and decomposition of two types of genetic data and fails three or higher types. These limitations inspire us to continue working in the future.

## Data Availability Statement

The original contributions presented in the study are included in the article/Supplementary Material, further inquiries can be directed to the corresponding author/s.

## Author Contributions

QD and YS designed the NMFNA method. QD and JS implemented and performed the experiments. QD, FL, YZ, and J-XL analyzed the experiment results and wrote the manuscript. All authors read and approved the final manuscript.

## Conflict of Interest

The authors declare that the research was conducted in the absence of any commercial or financial relationships that could be construed as a potential conflict of interest.

## Footnotes

1 https://cancergenome.nih.gov/

2 http://www.ncbi.nlm.nih.gov/geo/

3 https://david.ncifcrf.gov

4 https://github.com/xmao/kobas

5 http://www.genecards.org

## Supplementary Material

Supplementary Figure 1 Common genes of four GEO datasets.
