# Supplementary material for: NMFNA: A Non-negative Matrix Factorization Network Analysis Method for Identifying Modules and Characteristic Genes of Pancreatic Cancer
Source: Front Genet. 2021 Jul 22;12:678642. doi: 10.3389/fgene.2021.678642 (PMC8340025; doi:10.3389/fgene.2021.678642)

# Additional file

We suppose that the loss function of the objective function is non-increasing under the update rules in Equation (1), Equation (2), Equation (3) and Equation (4):

$(s_{11})_{kk}\leftarrow s_{11}\frac{(\mathbf{G}_{1}^{T}\mathbf{R}_{11}\mathbf{G}_{1})_{kk}}{(\mathbf{G}_{1}^{T}\mathbf{G}_{1}\mathbf{S}_{11}\mathbf{G}_{1}^{T}\mathbf{G}_{1})_{kk}}$, (1)

$(s_{22})_{kk}\leftarrow s_{22}\frac{(\mathbf{G}_{2}^{T}\mathbf{R}_{22}\mathbf{G}_{2})_{kk}}{(\mathbf{G}_{2}^{T}\mathbf{G}_{2}\mathbf{S}_{22}\mathbf{G}_{2}^{T}\mathbf{G}_{2})_{kk}}$, (2)

$(g_{1})_{ik}\leftarrow(g_{1})_{ik}\frac{(\alpha\mathbf{R}_{12}\mathbf{G}_{2}+2\mathbf{R}_{11}\mathbf{G}_{1}\mathbf{S}_{11}+2\lambda_{1}\mathbf{ZG}_{1})_{ik}}{(2\mathbf{G}_{1}\mathbf{S}_{11}\mathbf{G}_{1}^{T}\mathbf{G}_{1}\mathbf{S}_{11}+\alpha\mathbf{G}_{1}\mathbf{G}_{2}^{T}\mathbf{G}_{2}+2\lambda_{1}\mathbf{DG}_{1})_{ik}}$, (3)

$(g_{2})_{kj}\leftarrow(g_{2})_{kj}\frac{(\alpha\mathbf{R}_{12}^{T}\mathbf{G}_{1}+2\beta\mathbf{R}_{22}\mathbf{G}_{2}\mathbf{S}_{22}+\lambda_{2}\mathbf{ZG}_{2})_{kj}}{(\alpha\mathbf{G}_{2}\mathbf{G}_{1}^{T}\mathbf{G}_{1}+2\beta\mathbf{G}_{2}\mathbf{S}_{22}\mathbf{G}_{2}^{T}\mathbf{G}_{2}\mathbf{S}_{22}+\lambda_{2}\mathbf{DG}_{2})_{kj}}$. (4)

Meanwhile, when the elements $\left(g_{1}\right)_{ik}$,$\left(g_{2}\right)_{kj}$, $\left(s_{11}\right)_{kk}$ and $\left(s_{22}\right)_{ik}$ are stationary points, the loss function is constant. To prove that, we introduce the auxiliary function$\mathrm{G}$.$\mathrm{G}\left(h,h'\right)$is defined as an auxiliary function for the objective function $\mathrm{J}\left(h\right)$, when the following are satisfied:

$\mathrm{G}\left(h,h'\right)\geq\mathrm{J}\left(h\right) \forall h,\text{ }\mathrm{G}\left(h,h\right)=\mathrm{J}\left(h\right)$. (5)

Auxiliary functions have the following two properties:

Firstly, if $\mathrm{G}$ is an auxiliary function for $\mathrm{J}$, and $h^{\left(t+1\right)}=\mathrm{argmin}_{h}\mathrm{G}\left(h,h^{\left(t\right)}\right)$, then

$\mathrm{J}\left(h^{\left(t+1\right)}\right)\leq\mathrm{J}\left(h^{\left(t\right)}\right)$. (6)

The proof of Equation (6) is as follows: $\mathrm{J}\left(h^{\left(t+1\right)}\right)\leq\mathrm{G}\left(h^{\left(t+1\right)},h^{\left(t\right)}\right)\leq\mathrm{G}\left(h^{\left(t\right)},h^{\left(t\right)}\right)= \mathrm{J}\left(h^{\left(t\right)}\right)$ is firstly obtained from Equation (5) and Equation (6). Suppose there is a valid auxiliary function $\mathrm{G}_{\mathrm{J}}$ of the objective function. If the minimum updates rule for $\mathrm{G}_{\mathrm{J}}$ is equivalent toEquation (1),Equation (2), Equation(3) andEquation (4), the convergence of the objective function can be proved. Then, using $(\mathrm{J}_{\mathbf{S}_{11}})_{kk}$, $(\mathrm{J}_{\mathbf{S}_{22}})_{kk}$, $(\mathrm{J}_{\mathbf{G}_{1}})_{ik}$and $(\mathrm{J}_{\mathbf{G}_{2}})_{jk}$ to represent the elements $\left(\mathbf{G}_{1}\right)_{ik}\in\mathbf{G}_{1}$, $\left(\mathbf{G}_{2}\right)_{kj}\in\mathbf{G}_{2}$, $\left(\mathbf{S}_{11}\right)_{kk}\in\mathbf{S}_{11}$ and $\left(\mathbf{S}_{22}\right)_{ik}\in\mathbf{S}_{22}$, respectively. The partial derivative equation of the objective function can be inferred as follows:

$\text{（}\mathrm{J}_{\mathbf{S}_{11}})_{kk}^{'}=\left(\frac{\partial f}{\partial\mathbf{S}_{11}}\right)_{kk}=(-2\mathbf{G}_{1}^{T}\mathbf{R}_{11}\mathbf{G}_{1}+2\mathbf{G}_{1}^{T}\mathbf{G}_{1}\mathbf{S}_{11}\mathbf{G}_{1}^{T}\mathbf{G}_{1})_{kk}$, (7)

$(\mathrm{J}_{\mathbf{S}_{11}})_{kk}^{''}=\left(\frac{\partial^{2}f}{\partial\mathbf{S}_{11}^{2}}\right)_{kk}=(2\mathbf{G}_{1}^{T}\mathbf{G}_{1}\mathbf{G}_{1}^{T}\mathbf{G}_{1})_{kk}$, (8)

$\text{（}\mathrm{J}_{\mathbf{S}_{22}})_{kk}^{'}=\left(\frac{\partial f}{\partial\mathbf{S}_{22}}\right)_{kk}=(-2\mathbf{G}_{2}^{T}\mathbf{R}_{22}\mathbf{G}_{2}+2\mathbf{G}_{2}^{T}\mathbf{G}_{2}\mathbf{S}_{22}\mathbf{G}_{2}^{T}\mathbf{G}_{2})_{kk}$, (9)

$(\mathrm{J}_{\mathbf{S}_{22}})_{kk}^{''}=\left(\frac{\partial^{2}f}{\partial\mathbf{S}_{22}^{2}}\right)_{kk}=(2\mathbf{G}_{2}^{T}\mathbf{G}_{2}\mathbf{G}_{2}^{T}\mathbf{G}_{2})_{kk}$, (10)

$(\mathrm{J}_{\mathbf{G}_{1}})_{ik}^{'}=\frac{\partial f}{\partial\mathbf{G}_{1}}=(4\mathbf{G}_{1}\mathbf{S}_{11}\mathbf{G}_{1}^{T}\mathbf{G}_{1}\mathbf{S}_{11}-4\mathbf{R}_{11}\mathbf{G}_{1}\mathbf{S}_{11}+2\alpha\mathbf{G}_{1}\mathbf{G}_{2}^{T}\mathbf{G}_{2}-2\alpha\mathbf{R}_{12}\mathbf{G}_{2}+2\lambda_{1}\mathbf{LG}_{1}+\mathbf{\psi}_{3})_{ik}$, (11)

$(\mathrm{J}_{\mathbf{G}_{1}})_{ik}^{''}=\left(\frac{\partial^{2}f}{\partial\mathbf{G}_{1}^{2}}\right)_{ik}=-4(\mathbf{R}_{11})_{ii}(\mathbf{S}_{11})_{kk}+(12\mathbf{G}_{1}\mathbf{S}_{11}\mathbf{G}_{1}^{T}\mathbf{S}_{11}+2\alpha\mathbf{G}_{2}^{T}\mathbf{G}_{2}+2\lambda_{1}\mathbf{L})_{ik}$, (12)

$(\mathrm{J}_{\mathbf{G}_{2}})_{jk}^{'}=(\frac{\partial f}{\partial\mathbf{G}_{2}})_{jk}=\text{（}4\beta(\mathbf{G}_{2}\mathbf{S}_{22}\mathbf{G}_{2}^{T}\mathbf{G}_{2}\mathbf{S}_{22}-\mathbf{R}_{22}\mathbf{G}_{2}\mathbf{S}_{22}\text{）}+2\text{（}\alpha\mathbf{G}_{2}\mathbf{G}_{1}^{T}\mathbf{G}_{1}-\alpha\mathbf{R}_{12}\mathbf{G}_{1}+\lambda_{2}\mathbf{LG}_{2}\text{）}+\mathbf{\psi}_{4})_{jk}$, (13)

$(\mathrm{J}_{\mathbf{G}_{2}})_{jk}^{''}=\left(\frac{\partial^{2}f}{\partial\mathbf{G}_{1}^{2}}\right)_{jk}=-4\beta(\mathbf{R}_{22})_{jj}(\mathbf{S}_{22})_{kk}+(12\beta\mathbf{G}_{2}\mathbf{S}_{22}\mathbf{G}_{2}^{T}\mathbf{S}_{22}+2\alpha\mathbf{G}_{1}^{T}\mathbf{G}_{1}+2\lambda_{2}\mathbf{L})_{jk}$. (14)

Since the algorithm updates each element, the objective function is non-increasing, unless the elements $(\mathrm{J}_{\mathbf{S}_{11}})_{kk}$, $(\mathrm{J}_{\mathbf{S}_{22}})_{kk}$, $(\mathrm{J}_{\mathbf{G}_{1}})_{ik}$and $(\mathrm{J}_{\mathbf{G}_{2}})_{jk}$are non-increasing.

Secondly, the expansion items of $\mathrm{G}_{\mathrm{J}}\left(s_{11},\left(s_{11}\right)_{kk}^{t}\right)$, $\mathrm{G}_{\mathrm{J}}\left(s_{22},\left(s_{22}\right)_{kk}^{t}\right)$, $\mathrm{G}_{\mathrm{J}}\left(g_{1},\left(g_{1}\right)_{ik}^{t}\right)$ and $\mathrm{G}_{\mathrm{J}}\left(g_{2},\left(g_{2}\right)_{jk}^{t}\right)$ can be written as Equation (15), Equation (16), Equation (17) and Equation (18), which are regarded as auxiliary functions of the $\left(s_{11}\right)_{kk}$, $\left(s_{22}\right)_{kk}$, $\left(g_{1}\right)_{ik}$and $\left(g_{2}\right)_{jk}$, respectively:

$\begin{matrix} \mathrm{G}_{\mathrm{J}}\left(s_{11},\left(s_{11}\right)_{kk}^{t}\right)=(\mathrm{J}_{s_{11}})_{kk}\left(\left(s_{11}\right)_{kk}^{t}\right)+(\mathrm{J}_{s_{11}})_{kk}^{'}\left(\left(s_{11}\right)_{kk}^{t}\right)\left(s_{11}-\left(s_{11}\right)_{kk}^{t}\right) \\ +\frac{(\mathbf{G}_{1}^{T}\mathbf{G}_{1}\mathbf{S}_{11}\mathbf{G}_{1}^{T}\mathbf{G}_{1})_{kk}}{\left(s_{11}\right)_{kk}^{t}}\left(s_{11}-\left(s_{11}\right)_{kk}^{t}\right)^{2} \end{matrix}$, (15)

$\begin{matrix} \mathrm{G}_{\mathrm{J}}\left(s_{22},\left(s_{22}\right)_{kk}^{t}\right)=(\mathrm{J}_{s_{22}})_{kk}\left(\left(s_{22}\right)_{kk}^{t}\right)+(\mathrm{J}_{s_{22}})_{kk}^{'}\left(\left(s_{22}\right)_{kk}^{t}\right)\left(s_{22}-\left(s_{22}\right)_{kk}^{t}\right) \\ +\frac{(\mathbf{G}_{2}^{T}\mathbf{G}_{2}\mathbf{S}_{22}\mathbf{G}_{2}^{T}\mathbf{G}_{2})_{kk}}{\left(s_{22}\right)_{kk}^{t}}\left(s_{22}-\left(s_{22}\right)_{kk}^{t}\right)^{2} \end{matrix}$, (16)

$\begin{matrix} \mathrm{G}_{\mathrm{J}}\left(g_{1},\left(g_{1}\right)_{ik}^{t}\right)=(\mathrm{J}_{g_{1}})_{ik}\left(\left(g_{1}\right)_{ik}^{t}\right)+(\mathrm{J}_{g_{1}})_{ik}^{'}\left(\left(g_{1}\right)_{ik}^{t}\right)\left(g_{1}-\left(g_{1}\right)_{ik}^{t}\right) \\ +\frac{(-2\mathbf{R}_{11}\mathbf{G}_{1}\mathbf{S}_{11}+2\mathbf{G}_{1}\mathbf{S}_{11}\mathbf{G}_{1}^{T}\mathbf{G}_{1}\mathbf{S}_{11}+\alpha\mathbf{G}_{1}\mathbf{G}_{2}^{T}\mathbf{G}_{2}+\lambda_{1}\mathbf{LG}_{1})_{ik}}{\left(g_{1}\right)_{ik}^{t}}\left(g_{1}-\left(g_{1}\right)_{ik}^{t}\right)^{2} \end{matrix}$, (17)

$\begin{matrix} \mathrm{G}_{\mathrm{J}}\left(g_{2},\left(g_{2}\right)_{jk}^{t}\right)=(\mathrm{J}_{g_{2}})_{jk}\left(\left(g_{2}\right)_{jk}^{t}\right)+(\mathrm{J}_{g_{2}})_{jk}^{'}\left(\left(g_{2}\right)_{jk}^{t}\right)\left(g_{2}-\left(g_{2}\right)_{jk}^{t}\right) \\ +\frac{(-2\beta\mathbf{R}_{22}\mathbf{G}_{2}\mathbf{S}_{22}+2\beta\mathbf{G}_{1}\mathbf{S}_{22}\mathbf{G}_{2}^{T}\mathbf{G}_{2}\mathbf{S}_{22}+\alpha\mathbf{G}_{2}\mathbf{G}_{1}^{T}\mathbf{G}_{1}+\lambda_{2}\mathbf{LG}_{2})_{jk}}{\left(g_{2}\right)_{jk}^{t}}\left(g_{2}-\left(g_{2}\right)_{jk}^{t}\right)^{2} \end{matrix}$. (18)

The process of proof is as follows: we firstly obtained $\mathrm{G}_{\mathrm{J}}\left(s_{11},s_{11}\right)=(\mathrm{J}_{s_{11}})_{kk}\left(s_{11}\right)$, $\mathrm{G}_{\mathrm{J}}\left(s_{22},s_{22}\right)=(\mathrm{J}_{s_{22}})_{kk}\left(s_{22}\right)$, $\mathrm{G}_{\mathrm{J}}\left(g_{1},g_{1}\right)=(\mathrm{J}_{g_{1}})_{ik}\left(g_{1}\right)$ and $\mathrm{G}_{\mathrm{J}}\left(g_{2},g_{2}\right)=(\mathrm{J}_{g_{2}})_{jk}\left(g_{2}\right)$ according to the above description. Then, Taylor series expansion of the auxiliary function are defined as follows:

$\begin{matrix} (\mathrm{J}_{s_{11}})_{kk}\left(s_{11}\right)=(\mathrm{J}_{s_{11}})_{kk}\left(\left(s_{11}\right)_{kk}^{t}\right)+(\mathrm{J}_{s_{11}})_{kk}^{'}\left(\left(s_{11}\right)_{kk}^{t}\right)\left(s_{11}-\left(s_{11}\right)_{kk}^{t}\right) \\ +\frac{1}{2}(\mathrm{J}_{s_{11}})_{kk}^{''}\left(\left(s_{11}\right)_{kk}^{t}\right)\left(s_{11}-\left(s_{11}\right)_{kk}^{t}\right)^{2} \end{matrix}$, (19)

$\begin{matrix} (\mathrm{J}_{s_{22}})_{kk}\left(s_{22}\right)=(\mathrm{J}_{s_{22}})_{kk}\left(\left(s_{22}\right)_{kk}^{t}\right)+(\mathrm{J}_{s_{22}})_{kk}^{'}\left(\left(s_{22}\right)_{kk}^{t}\right)\left(s_{22}-\left(s_{22}\right)_{kk}^{t}\right) \\ +\frac{1}{2}(\mathrm{J}_{s_{22}})_{kk}^{''}\left(\left(s_{22}\right)_{kk}^{t}\right)\left(s_{22}-\left(s_{22}\right)_{kk}^{t}\right)^{2} \end{matrix}$, (20)

$\begin{matrix} (\mathrm{J}_{g_{1}})_{ik}\left(g_{1}\right)=(\mathrm{J}_{g_{1}})_{ik}\left(\left(g_{1}\right)_{ik}^{t}\right)+(\mathrm{J}_{g_{1}})_{ik}^{'}\left(\left(g_{1}\right)_{ik}^{t}\right)\left(g_{1}-\left(g_{1}\right)_{ik}^{t}\right) \\ +\frac{1}{2}(\mathrm{J}_{g_{1}})_{ik}^{''}\left(\left(g_{1}\right)_{ik}^{t}\right)\left(g_{1}-\left(g_{1}\right)_{ik}^{t}\right)^{2} \end{matrix}$, (21)

$\begin{matrix} (\mathrm{J}_{g_{2}})_{jk}\left(g_{2}\right)=(\mathrm{J}_{g_{2}})_{jk}\left(\left(g_{2}\right)_{jk}^{t}\right)+(\mathrm{J}_{g_{2}})_{jk}^{'}\left(\left(g_{2}\right)_{jk}^{t}\right)\left(g_{2}-\left(g_{2}\right)_{jk}^{t}\right) \\ +\frac{1}{2}(\mathrm{J}_{g_{2}})_{jk}^{''}\left(\left(g_{2}\right)_{jk}^{t}\right)\left(g_{2}-\left(g_{2}\right)_{jk}^{t}\right)^{2} \end{matrix}$. (22)

Next, $\mathrm{G}_{\mathrm{J}}\left(s_{11},s_{11}\right)\geq(\mathrm{J}_{s_{11}})_{kk}\left(s_{11}\right)$, $\mathrm{G}_{\mathrm{J}}\left(s_{22},s_{22}\right)\geq(\mathrm{J}_{s_{22}})_{kk}\left(s_{22}\right)$, $\mathrm{G}_{\mathrm{J}}\left(g_{1},g_{1}\right)\geq(\mathrm{J}_{g_{1}})_{ik}\left(g_{1}\right)$and $\mathrm{G}_{\mathrm{J}}\left(g_{2},g_{2}\right)\geq(\mathrm{J}_{g_{2}})_{jk}\left(g_{2}\right)$ need to be satisfied. According toEquation (14) andEquation (18), $\mathrm{G}_{\mathrm{J}}\left(s_{11},s_{11}\right)\geq(\mathrm{J}_{s_{11}})_{kk}\left(s_{11}\right)$ can be expanded as follows:

$\frac{(\mathbf{G}_{1}^{T}\mathbf{G}_{1}\mathbf{S}_{11}\mathbf{G}_{1}^{T}\mathbf{G}_{1})_{kk}}{\left(s_{11}\right)_{kk}^{t}}\geq(\mathbf{G}_{1}^{T}\mathbf{G}_{1}\mathbf{G}_{1}^{T}\mathbf{G}_{1})_{kk}$. (23)

Since $(\mathbf{G}_{1}^{T}\mathbf{G}_{1}\mathbf{S}_{11}\mathbf{G}_{1}^{T}\mathbf{G}_{1})_{kk}=\sum_{a=1}^{k} \left(s_{11}\right)_{ia}^{t}(\mathbf{G}_{1}^{T}\mathbf{G}_{1}\mathbf{G}_{1}^{T}\mathbf{G}_{1})_{ak}$ and $\sum_{a=1}^{k} \left(s_{11}\right)_{ia}^{t}(\mathbf{G}_{1}^{T}\mathbf{G}_{1}\mathbf{G}_{1}^{T}\mathbf{G}_{1})_{ak}\geq\left(s_{11}\right)_{kk}^{t}(\mathbf{G}_{1}^{T}\mathbf{G}_{1}\mathbf{G}_{1}^{T}\mathbf{G}_{1})_{kk}$, so we obtained $\mathrm{G}_{\mathrm{J}}\left(s_{11},s_{11}\right)\geq(\mathrm{J}_{s_{11}})_{kk}\left(s_{11}\right)$. Similarly, we can also obtained $\mathrm{G}_{\mathrm{J}}\left(s_{22},s_{22}\right)\geq(\mathrm{J}_{s_{22}})_{kk}\left(s_{22}\right)$, $\mathrm{G}_{\mathrm{J}}\left(g_{1},g_{1}\right)\geq(\mathrm{J}_{g_{1}})_{ik}\left(g_{1}\right)$ and $\mathrm{G}_{\mathrm{J}}\left(g_{2},g_{2}\right)\geq(\mathrm{J}_{g_{2}})_{jk}\left(g_{2}\right)$. After the above derivation, it can be proofed that the auxiliary functions $(\mathrm{J}_{\mathbf{S}_{11}})_{kk}$, $(\mathrm{J}_{\mathbf{S}_{22}})_{kk}$, $(\mathrm{J}_{\mathbf{G}_{1}})_{ik}$and $(\mathrm{J}_{\mathbf{G}_{2}})_{jk}$of the updated formulas Equation (1), Equation(2), Equation(3) and Equation (4) are non-increasing. At the same time, the proof of the first property is completed. In conclusion, the derivation shows that the loss function of the objective function is convergent.

# Figure

Figure. Common genes of four GEO datasets.


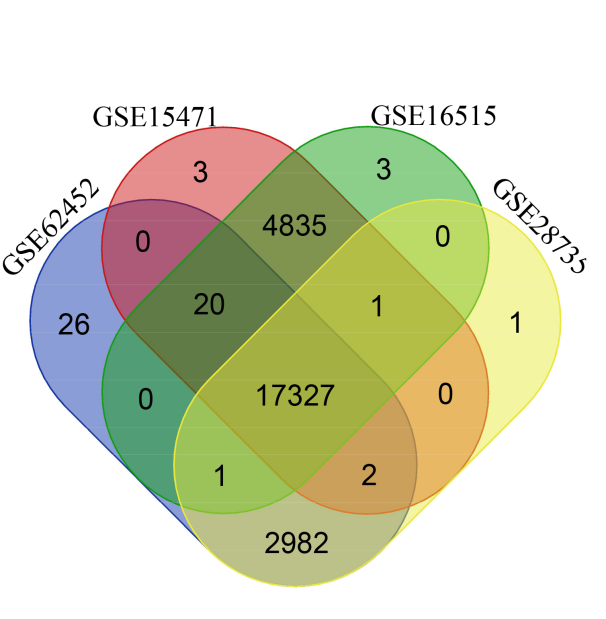

Supplement: Supplementary Figure 1 — Common genes of four GEO datasets. [file Data_Sheet_1.docx]
